# Initiatives supporting evidence informed health system policymaking in Cameroon and Uganda: a comparative historical case study

**DOI:** 10.1186/s12913-014-0612-3

**Published:** 2014-11-29

**Authors:** Pierre Ongolo-Zogo, John N Lavis, Goran Tomson, Nelson K Sewankambo

**Affiliations:** Health Policy and Knowledge Translation Doctoral Program, Makerere University College of Health Sciences, Kampala, Uganda; Department of Clinical Epidemiology and Biostatistics, McMaster University, 1280 Main Street West, CRL 209, Hamilton, Ontario L8S 4 K1 Canada; Departments of Learning, Informatics, Management, Ethics and Public Health Sciences, Karolinska Institutet, Stockholm, Sweden; Centre for Development of Best Practices in Health, Central Hospital Yaoundé, University of Yaoundé, Yaoundé, Cameroon

**Keywords:** Evidence informed health system policymaking, Knowledge translation platform, Health systems, Low- and middle- income countries, Governance, Comparative case study, Cameroon, Uganda

## Abstract

**Background:**

There is a scarcity of empirical data on institutions devoted to knowledge brokerage and their influence in Africa. Our objective was to describe two pioneering Knowledge Translation Platforms (KTPs) supporting evidence informed health system policymaking (EIHSP) in Cameroon and Uganda since 2006.

**Methods:**

This comparative historical case study of Evidence Informed Policy Network (EVIPNet) Cameroon and Regional East African Community Health Policy Initiative (REACH-PI) Uganda using multiple methods comprised (i) a descriptive documentary analysis for a narrative historical account, (ii) an interpretive documentary analysis of the context, profiles, activities and outputs inventories and (iii) an evaluative survey of stakeholders exposed to evidence briefs produced and policy dialogues organized by the KTPs.

**Results:**

Both initiatives benefited from the technical and scientific support from the global EVIPNet resource group. EVIPNet Cameroon secretariat operates with a multidisciplinary group of part-time researchers in a teaching hospital closely linked to the ministry of health. REACH-PI Uganda secretariat operates with a smaller team of full time staff in a public university. Financial resources were mobilized from external donors to scale up capacity building, knowledge management, and linkage and exchange activities. Between 2008 and 2012, twelve evidence briefs were produced in Cameroon and three in Uganda. In 2012, six rapid evidence syntheses in response to stakeholders’ urgent needs were produced in Cameroon against 73 in Uganda between 2010 and 2012. Ten policy dialogues (seven in Cameroon and three in Uganda) informed by pre-circulated evidence briefs were well received. Both KTPs contributed to developing and testing new resources and tools for EIHSP. A network of local and global experts has created new spaces for evidence informed deliberations on priority health policy issues related to MDGs.

**Conclusion:**

This descriptive historical account of two KTPs housed in government institutions in Africa illustrates how the convergence of local and global factors and agents has enabled in-country efforts to support evidence-informed deliberations on priority health policy issues and lays the ground for further work to assess their influence on the climate for EIHSP and specific health policy processes.

## Background

Poor access to health interventions and poor performance of health systems are consistently an issue of concern for national and global stakeholders as we approach the year 2015, and many predict the failure to achieve the targets set for health Millennium Development Goals - MDGs - particularly in sub Saharan countries [1-3]. Fostering evidence informed health system policymaking (EIHSP) in low- and middle-income countries (LMICs) has become a priority for the United Nations and development agencies striving to bridge the “know-do” gap that undermines progress towards the health MDGs. As a consequence, several agencies are providing financial support to tens of initiatives worldwide in that regard [4,5].

Knowledge Translation Platforms (KTPs) are such an initiative that brings together policymakers, researchers and other stakeholders including civil society for evidence informed deliberations on health priorities. KTPs are conceived as knowledge brokering enterprises building from the integrated model for linking research to policy [6-9]. Examples in Africa are Evidence Informed Policy Network (EVIPNet) and the Regional East African Community - Health Policy Initiative (REACH-PI) together involving twelve countries [10,11] with EVIPNet Cameroon and REACH-PI Uganda being amongst the most active [12]. The premise of such efforts is that the use of research evidence for health system policymaking will yield positive public health and social impacts [13].

While several case-studies have gathered evidence on the impact of health technology assessment units and government support units in high-income countries [14,15] and few case-studies of embedded knowledge translation strategies within research to policy projects in LMICs [16], there is a scarcity of empirical data on institutions devoted to knowledge brokerage and their influence [17-19]. Boaz and colleagues [20],concluding a systematic review, called for the development of new conceptual frameworks and methods to orient future evaluations of interventions designed to promote research use, including knowledge brokers, networks, and linkage and exchange programmes.

The lack of systematic documentation of the KTPs in LMICs prevents learning from these social innovations in countries synonymous with scarcity. The objective of this paper is to describe and interpret the history, the infrastructure, the activities, and the outputs of two pioneering KTPs in Cameroon and Uganda established since 2006.

## Methods

We conducted a comparative historical case study of two KTPs within their contexts using multiple methods [21]. EVIPNet Cameroon housed at the Central Hospital, Yaoundé and REACH-PI Uganda housed at Makerere University College of Health Sciences, Kampala were identified for their exemplarity as pioneers in Central and Eastern Africa during the period 2001–2012. This post Millennium Summit timeframe was retained in order to investigate two six-year periods before and after the launching of both initiatives in 2006. The investigation comprised (i) a descriptive documentary analysis to provide a narrative historical account, (ii) an interpretive documentary analysis of the context and the profile/activities and outputs inventories and (iii) an evaluative survey of stakeholders exposed to evidence briefs for policy and invited to policy dialogues. The authors stand as insiders intervening either as policymaker (POZ), knowledge broker leading a KTP secretariat since its inception (POZ, NKS) or investigator in the Supporting the Use of Research Evidence for policy in African health systems – SURE research project (POZ, NKS, JNL and GT) [www.global.evipnet.org/sure] and Knowledge Translation Platforms Evaluation– KTPE research project (POZ, NKS, JNL)[22]. As a group of authors with different levels of engagement in the KTP activities, we strived to maintain as much neutrality and objectivity by combining different sources of data, online discussions on and several iterations of the draft manuscript.

### Document review

We conducted a qualitative descriptive and interpretive archival review of both initiatives. All the available documents were requested from the KTPs’ secretariats, the research coordinators of KTPE project at McMaster University in Hamilton, Canada and SURE project at the Norwegian Knowledge Centre for Health Sciences in Oslo, Norway. We searched the websites of EVIPNet (www.global.evipnet.org), EVIPNet Cameroon (www.cdbph.org), and the Uganda clearinghouse for health policy and systems research (www.uchpsr.org) for any relevant documents or activities. We equally conducted a structured documentary review of poverty reduction/eradication strategic papers and health sector strategic plans produced in Cameroon and Uganda during the period 2001–2012 to capture the political, social and economic contexts and salient features of both health systems. These documents were obtained from the respective ministries of health (Table 1). We extracted relevant data featuring the contexts, the institutional arrangements, the activities and outputs of KTPs.

**Table 1:** **Data sources**

**Cameroon**	**Years**	**Uganda**
Health sector strategic plan 2001-10	2001	
Poverty reduction strategic paper 2003-10	2003	
	2004	Poverty Eradication Action Plan 2004/5-2007/8
	2005	Health sector strategic plan II 2005/6-2009/10
Letter of Intent to global EVIPNet	2006	REACH Prospectus – Uganda
GHLA Grant application	2007	
AHPSR Grant application	2008	IRCI Grant application – Uganda
IDRC grant application EVIPNet	2009	IDRC grant application
Health sector strategic plan 2001-15	2009	National Development Plan 2010/11-2014/15
		Health sector strategic and investment plan 2010/11-2014/15
Growth and employment strategic paper 2010-20	2010	
SURE annual reports	2010- 2012	SURE annual reports
IDRC grant reports	2010- 2012	IDRC grant reports
www.cdbph.org	n/a	www.uchpsr.org
www.global.evipnet.org	n/a	www.global.evipnet.org
EIHP International Forum Report	2012	EIHP International Forum Report
EVIPNet strategic plan	2012	EVIPNet strategic plan
EVIPNet Africa 2006–2012 Lessons learned	2013	EVIPNet Africa 2006–2012 Lessons learned
Evaluative survey of evidence briefs and policy dialogues	2009-2012	Evaluative survey of evidence briefs and policy dialogues

### Evaluative survey

In both countries, policymakers, researchers and other stakeholders likely to be involved in or affected by policy decisions on the issue addressed by an evidence brief (policy brief) were invited to a deliberative dialogue (policy or stakeholder dialogue). All dialogue participants were surveyed about the evidence brief that was pre-circulated for the dialogue and about the dialogue itself. Seven evidence briefs and five dialogues were concerned in Cameroon. Three evidence briefs and two policy dialogues were concerned in the case of Uganda. The questionnaires available both in French and English were developed as part of the KTPE study. Each questionnaire comprised three or four sections to depict how helpful each of the features of the brief/dialogue were, how well the brief/dialogue achieved its intended purpose, items based on theory of planned behaviour constructs, and questions about respondents’ professional experiences. Further details on the instruments can be accessed online at http://www.researchtopolicy.org/KTPEs/KTPE-overview.

The coding of the features of the brief/dialogue based on the electronic copies of the dialogue summary and/or report was checked with core members of each KTP secretariat. Descriptive statistics were used to examine respondents’ overall assessments of brief/dialogue and their features and to profile the assessments of each feature of the brief/dialogue, each of the brief and the dialogue as a whole, and respondents’ intentions to act on what they had learned.

## Analytical framework

To systematically describe the KTPs, we elaborated an analytical framework (Table 2) from a purposive review of writing including frameworks, concepts and theories pertaining to knowledge brokerage and the integrated model for knowledge translation [6-9]. The latter is underpinned by social learning theory and planned behaviour change model geared at addressing barriers and facilitators to research use by policymakers within the “two-community” thesis [23]. Several scholars have explained the poor use of research evidence into policymaking by the differences of cultures across the research community and the policy community thus establishing the foundations of the knowledge brokerage, and linkage and exchange models [6-9,24-28]. From the political sciences, we draw from the health policy analysis triangle [29,30], policy networks [31-33] and the critical drivers of policymaking – institutions, interests, ideas and external factors [34,35].

**Table 2:** **KTP Analytical Framework**

**Functions**	**Domains**	**Activities**	**Outputs**	**Targets of influence**
Capacity Building	Research and evidence production	Workshops to conduct relevant research and prepare evidence syntheses	Skilled individuals	Researchers
			Resources and tools	
	Linking evidence to policy	Workshops to demand and access evidence resources	Skilled individuals	Stakeholders
			Resources and tools	Policymakers
	Evaluation	Ongoing monitoring	Annual reports	Institutions, interests, ideas and external factors
			Lessons learnt	
	Sustainability	Grant applications	Meeting reports	
		Advocacy meetings	Grants	
		Doctoral studies program		
Knowledge Management	Planning	Priority setting exercises	Lists of health priority issues and evidence gaps	Research content
				Research processes
	Research production and synthesis	Synthesizing evidence	Systematic reviews	Policy content
		Summarizing evidence	Evidence briefs and summaries	Policy processes
				Ideas
	Dissemination	Maintaining a clearinghouse	Resources, tools and evidence	Interests, ideas
		Conference participation	Abstract books	
Linkage and Exchange	Linkage	Priority setting exercises	Meeting reports	Interests, ideas
		Facilitating user-pull		Policy processes
	Exchange	Organizing deliberative dialogues	Dialogue reports	Research processes
				External actors

The framework combines the three functions of a knowledge brokering enterprise [28] with the domains and elements to assess country efforts to link evidence to action [9] as well as activities and outputs deemed to influence the policy context, process and content [29,30] and the critical drivers of policymaking and to eventually intersect with contextual factors such as political and health systems and policy networks. We described and analyzed the health systems according to their governance, financial and delivery arrangements, as well as health technology provisions [35]. We used the interpretive constant comparison of KTPs within their contexts to highlight similarities and differences.

The study was approved by the ethics review board at the Makerere University College of Health Sciences and the Ministry of Health in Cameroon.

## Results

### Study context

Cameroon and Uganda political systems are marked by their presidential regimes strongly anchored in traditional ruling systems bolstering the ethnic diversity with 220 and 56 ethnic groups respectively. The Head of State in each country has been in office since the 1980’s. The Parliaments are dominated by a large majority from the Head of State’s political party and technocrats play a pivotal role during health policymaking. The thrust of development policies has been the achievement of MDGs following the Millennium Summit and the African Union resolutions to speed up health investments and align them with health MDGs targets with both countries eligible for grants from a diversity of global health initiatives. Efforts were engaged to strengthen national health research systems leading to establishing a division in charge of health research in the ministry of health in Cameroon since 2002 and increasing financial support to Uganda National Health Research Organization (UNHRO) since 2008.

Since the mid 1990’s, health decentralization was initiated in both countries to align with the health district framework established by the African Regional Office of WHO. The tiered health systems are mixed; state owned health services coexist with private health facilities operating in poorly regulated environments. The ministry of health is the overarching health authority in addition to the inter-sectoral governing bodies of priority health programs established in response to global health initiatives (e.g.; expanded programme of immunization, control programs for AIDS, tuberculosis and malaria, reproductive health, neglected tropical diseases, etc.). The major changes observed include: i)the abolition of user fees in Uganda in 2001 and the promotion of community based health insurance in Cameroon since 2004; ii) the tangible efforts towards actual decentralization of health authority to provincial/regional and district authorities in both countries starting in 2001; iii) the promotion of universal access to HIV/AIDS care including antiretroviral therapy since 2003; iv) the universal access to malaria control interventions since 2002 and; v) the scaling up of reproductive health programs in line with the African Union’s campaign to accelerate the reduction of maternal mortality in 2009.

Table 3 summarizes the political and health systems and main indicators of health MDGs. While Cameroon ranked as lower middle income and Uganda as low income, the maternal mortality ratio (MDG 5) has worsened in the former while improving in the latter. Neither country will reach the health MDGs targets by 2015.

**Table 3:** **Cameroon and Uganda political and health systems**

	**Cameroon**		**Uganda**	
	**2001-06**	**2007-12**	**2001-06**	**2007-12**
**Political system features**				
Political regime	Presidential regime with the same President in office since the 1980’s. Prime Ministers are designated by the President. Traditional chiefdoms.			
Parliament	Large majority		Majority	
Leadership in the Ministry of Health	Two Ministers with the same Secretary of State in office. Three permanent secretaries in office and few changes of directors.		Three Ministers, three Director General and changes of high ranking civil servants in health policy and planning units	
**Tiered health system features**				
Health system governance arrangements	National ministry of health + inter-sectoral governing bodies for public health programmes. 10 provincial delegations and 143 districts with dialogue structures poorly functional.	National ministry of health + inter-sectoral governing bodies for public health programmes.	National ministry of health + inter-sectoral governing bodies for public health programmes.12 regional directions and 87 districts. Dialogue structures linked to different levels of local governments.	
		10 regional delegations and 178 districts with municipal leaders holding leadership positions in health district management boards.		
Health financial arrangements	User fees under a fee-for-service scheme in government owned facilities. The Government raise some funds from the general tax system and overseas development aid. Civil servants are paid by the central government but also receive bonus based on user fees. Private clinics operate under a poorly regulated fee-for-service scheme.	User fees under a fee-for-service scheme. 98% out of pocket payments. Despite a national strategy to promote community-based health insurance, coverage is below 2%. Rising petty corruption in state owned facilities.	Abolition of the user-for-service scheme in 2001 in government owned facilities. Civil servants are paid by the central government.	
Service delivery arrangements	Community health volunteers provide some benevolent primary health care services. Free preventive services in government health facilities. Private clinics operate under a fee-for-service scheme and pharmacies. Faith based and not for profit NGO health facilities operate under a subsidized fee-for-service scheme. Traditional healers and informal health facilities.			
Technologies, medicines and vaccines	A national procurement system for essential and generic medicines coexists with dedicated procurement systems for vertical priority health programs (vaccines, ART). Private medicines wholesalers operate under a poorly regulated environment in which drugs prices are free. Private medical equipment firms.			
**MDGs Indicators (from UNDP, 2011)**				
Population (millions)	18.055	19.522	20.9	32.71
MDG 4: under five mortality ratio/1000	148	136	137	115
MDG5: maternal mortality ratio/100000	669	780	510	430
MDG 6: HIV prevalence/1000	66	53	64	65
MDG6: tuberculosis prevalence/100000	270	191	304	209
MDG6: malaria mortality rate/100000	116	19	NA	16

### EVIPNet Cameroon and REACH-PI Uganda

The KTP secretariats are housed within a government entity, a teaching hospital closely linked to the ministry of health in Cameroon and a public university in case of REACH-PI Uganda. Since the beginning, each secretariat is led by the same local champion linked with global ‘evidence to policy’ specialists. Issue networks of policymakers, researchers and other stakeholders were established around priority topics (e.g.; reproductive health, governance for health district development, health financing, malaria control, human resources for health, etc.). The intersection with global funding opportunities for health, the formal and informal connections between the KTP secretariats and national and international players influenced priority setting exercises and resources mobilization to support KTPs operations.

### Historical account

Table 4 outlines a comparative historical account in relation to global focusing events. EVIPNet Cameroon can be traced from the creation of a division of health operations research in the ministry of health in 2002 whose mission includes linking research and action. The division responded to a call for letters of intent by the WHO which led to its establishment as the KTP secretariat in May 2006 before its relocation in June 2008 at the centre for the development of best practices in health at Central Hospital Yaoundé. REACH-PI Uganda came to existence through a longer incubation period starting in December 2001 with the Lake Duluti regional consultation under the auspices of the East African Health Research Council and concluded in December 2006. As a consequence of the regional consultation, several activities to link health research and action were conducted such as the successful completion of Tanzania Essential Health Interventions Project in 2003 and national workshops in 2004 and 2005 in Kenya, Tanzania and Uganda. The UNHRO agreed to have the Makerere University College of Health Sciences establish the Uganda REACH country node which marked the beginning of the Uganda KTP which has also served as the base for Makerere University’s participation in the SURE project.

**Table 4:** **Historical account of the KTPs development**

**Year**	**Global focusing event**	**Cameroon**	**Uganda**
2001	United Nations Organization launches the Millennium Development Goals (MDGs) including three health MDGs	Validation of the health sector strategic plan 2001-2015	Duluti Lake regional consultation convened by the MoH Tanzania to discuss gaps in research-policy-practice within the East African Community. Recommendation to approach Canada IDRC for support.
2002		A division of health operations research is established in the ministry of health	
2003	Completion of the TEHIP research project in Tanzania	A Director of the division of health operations research is appointed	Efforts to structure national knowledge translation activities following the consultation led by the National Institute of Medical Research Tanzania
2004	WHO AFRO Regional Committee endorses the “Roadmap for accelerating the attainment of MDGs 4 & 5. WHO Report “Knowledge for better health” highlights the need for enhanced knowledge translation efforts. Mexico Ministerial Summit on health research ends with a declaration calling for action.	Cameroon represented at the Mexico Ministerial Summit. Cameroon and COHRED launch collaboration for research priority setting and developing a national research policy. Establishment of an inter-sectoral consultative commission for health research	Uganda represented at the Mexico Ministerial Summit.
			Canada IDRC provides resources for the KTP work especially the concept development, the preparation of country cases studies and national workshops in Kenya, Tanzania and Uganda
2005	WHA resolution on Evidence Informed Policy Network (EVIPNet).	Creation of thematic research-to-policy groups for HIV-AIDS, malaria, tuberculosis and social sciences under the lead of the Division of health operations research. Conduct of the Health Research System Analysis in collaboration with Research Policy and Cooperation department at the WHO headquarter in Geneva	Country design workshops on the need, the institution, the function, autonomy, resources etc. Regional design workshop endorsed by the EAC Ministers of health. Funding proposal to support REACH-PI under EAC in Arusha. Launch of the Health Sector Strategic Plan 2005/06-09/10
2006	EVIPNet Africa launched in Brazzaville followed by a call for letters of intent for planning grants	Launch of the EVIPNet Cameroon with its secretariat housed in the division of health operations research. The initial priorities are malnutrition and non communicable diseases	Official launch of REACH-PI under the East Africa Health Research Council housed in Arusha
	Global Forum for Health Research meeting in Cairo with a focus on research partnership		Advocacy and resource mobilisation activities
	International Dialogue on EIHP in Thailand		
2007	SURE proposal development workshop to be submitted to the EC- FP7 (Oslo – Norway)	EVIPNet Cameroon receives a planning grant from WHO-HQ. Mid-term evaluation of the Health Sector Strategic Plan. EVIPNet Africa steering group is established	Financial support from IDRC, Swiss Tropical Institute and the Alliance for Health Policy and Systems Research (AHPSR) to REACH-PI. Evidence brief on male circumcision
2008	EVIPNet workshop on preparing policy briefs to scale up access to ACT Addis Ababa – Ethiopia	Preparation of the policy brief on scaling up access to ACT. Global Health Leadership Award(GHLA) from Canada Global Health Research Initiative to establish a knowledge brokerage unit, the Centre for the Development of Best Practices in Health – CDBPH at the Yaoundé Central Hospital to serve as EVIPNet Cameroon secretariat	Recruitment of a scientist/knowledge broker for REACH-PI in Arusha. Preparation of the policy brief on scaling up access to ACT. Uganda National Health Research Organization reinforces its knowledge translation activities.
	Bamako Ministerial Summit calling for a new impetus for knowledge translation. EC-FP7 selects SURE project for funding		
2009	African Union Conference of Ministers in Ethiopia launches the “campaign for accelerated reduction of maternal mortality in Africa”. IDRC awards an International Research Chair grant to support knowledge translation in Africa and for EVIPNet Africa. EVIPNet workshop in Paris. IJHTA published policy briefs on scaling up access to ACT. SUPPORT tools for EIHP.	Alliance for Health Policy and Systems Research awarded a grant to CDBPH to support the transition towards a Health SWAp to produce four evidence briefs on governance, health financing, and malaria control and health information system and to organize two policy dialogues on health financing and malaria control. Researchers attended capacity building workshops in Kampala	The Office of the Principal at Makerere University College of Health Sciences in Kampala is designated to host and manage the SURE grant on behalf REACH-PI. First SURE annual meeting and workshops to build capacity for EIHP. KTPE workshop on evaluation of knowledge translation platforms. The NOKC provided technical assistance to MUCHS to establish the SURE project
2010	The UN MDG report suggests goals 4 and 5 will not be met in Cameroon and Uganda. SURE annual meeting in Lusaka. First Global Symposium on Health Systems Research in Montreux- Switzerland with several sessions on EIHP. EVIPNet Africa call for applications on innovative strategies for EIHP	Priority setting for SURE project – more details in Table 6	Second National Health Policy, and Health Sector Strategic Plan 2010/11-2014/15
		Implementation of SURE project and AHPSR ID49 grant	
		Policy brief on governance for health district development	Implementation of SURE project – more details in Table 6
		CDBPH wins an EVIPNet grant to support capacity building for civil society and media on EIHP	Launch of the Rapid Response Service
			EVIPNet grant to support the Uganda clearinghouse
		Capacity building workshop to conduct Cochrane systematic reviews	IRCI research seminar
2011	SURE annual meeting in Maputo. Publication of the workbook for health systems guidance and the PLoS series on health systems guidance	Implementation of SURE project – more details in Table 6. The NOKC provided technical assistance to CDBPH. Co-application for a DFID grant onto support Effective Health Care Research with Stellenbosch University	Implementation of SURE project – more details in Table 6Implementation of EVIPNet grant
			IRCI Knowledge translation workshop
2012	International Forum on EIHP in LMIC Addis Ababa – Ethiopia. 2^nd^Global Symposium on HSR in Beijing – China	Implementation of SURE project – more details in Table 6	Implementation of SURE project – more details in Table 6
		IRCI Knowledge translation workshop	
		Launch of the Rapid Response Service	

### KTP institutional arrangements

Table 5 outlines the KTPs’ infrastructure. EVIPNet-Cameroon secretariat has been operating with a multidisciplinary group of part-time researchers and research assistants (e.g.; public health, economy, anthropology, sociology, epidemiology, clinical sciences) trained as brokers. While two scientists have remained engaged the whole time, a turn-over was noted amongst researchers and assistants. REACH-PI Uganda has been operating with a smaller group of full time staff of public health experts trained as brokers. A social scientist trained as broker left after 12 months and the number of brokers went from one to six between 2009 and 2012. The initial stakeholder analyses during grant preparation laid the groundwork for participatory priority setting exercises and validation of the respective programs of work thus creating the enabling environment for mutually beneficial exchange amongst knowledge brokers, policymakers, researchers, and other stakeholders. Both KTPs were established as demonstration projects informed by existing theoretical frameworks and were guided by a monitoring and evaluation framework that has enabled this description. They were conceived as problem solving enterprises, operating under the “learning through doing” principle. The same technical and scientific support from the EVIPNet resource group was provided to both initiatives for their operations including grant writing, developing and testing new resources and tools for EIHP. A vibrant collaboration was established with the McMaster Health Forum at McMaster University, the Norwegian Knowledge Centre for health services and the South African Cochrane Centre in Cape Town, South Africa (SACC). Several visits of scientists and knowledge brokers were organized across countries and in both directions.

**Table 5:** **KTP institutional arrangements**

**Characteristics**	**Cameroon**	**Uganda**
Goal	To build sustainable capacities for EIHP for better health in central Africa	To improve people’s health and health equity in East Africa through more effective use and application of knowledge to strengthen health policy and practice
Mission	To create human capacity and resources to create, demand and better use research syntheses for health improvement	To access, synthesize, package and communicate evidence required for policy and practice and for influencing policy relevant research agendas for improved population health and health equity
Governance arrangements	A research unit within the Yaoundé Central Hospital a teaching hospital closely linked with the Ministry of Public Health.	Research unit - Office of the Principal at the College of Health Sciences Makerere University Kampala, a public university working closely with the Uganda National Health Research Organization
	Issue-related Ad hoc steering group	
Stakeholders – audience	Researchers – policymakers – leaders of civil society representatives – journalists – development agencies – clinicians – senior officials from the ministry of health – hospital and program managers – students	Researchers – policymakers – leaders of civil society representatives – journalists – development agencies - senior officials from the ministry of health – hospital and program managers – students
Secretariat	One leading researcher, several part time researchers and short term research assistants	Stable supervisor, one research officer, full time assistant researchers and volunteers
International partnerships	AHPSR; CCGHR; CHSRF; NOKC; McMaster University; Stellenbosch University; WHO-EVIPNet	AHPSR; CCGHR; Karolinska Institutet; NOKC; McMaster University; WHO-EVIPNet
Sources of funding	WHO-EVIPNet; GHRI-GHLA; EC - FP7 SURE; AHPSR – ID49; DFID; IDRC; Cameroon Government	IDRC; AHPSR; Swiss Tropical Institute; WHO-EVIPNet; EC - FP7 SURE; Uganda Government
Estimated amount of funds received 2006–2012 ($ US)	720,000	The change of the hosting institution prevented us to have exhaustive figures on the whole period.

AHPSR: Alliance for Health Policy and Systems Research; CCGHR: Canadian Coalition for Global Health Research; CHSRF: Canada Health Services Research Foundation; DFID: United Kingdom Department for International Development; EU-FP7 SURE: European Union – Framework Program 7 Supporting the Use of Research Evidence for policy in African health systems; GHRI: Canada Global Health Research Initiative; IDRC: Canada International Development Research Centre; NOKC: Norwegian Knowledge Centre for health services; UNHRO: Uganda National Health Research Organization.

The initial funding in Cameroon was obtained from the global EVIPNet secretariat and the Canada’s Global Health Research Initiative through a Global Health Leadership Award. The initial funding for REACH-PI EAC was obtained from IDRC, the Swiss Tropical Institute and the Alliance for Health Policy and Systems Research (AHPSR). For the remaining period, financial resources for both KTPs were obtained from IDRC, the European Commission Seventh Framework Program (EC-FP7), the global EVIPNet secretariat and AHPSR. National governments provided mainly in-kind support. The EC-FP7 funded the five-year SURE project which was instrumental for building capacity, developing and testing resources and tools for EIHP. The Canadian Institutes for Health Research funded the KTPE. The estimated annual budget has varied from $US 40,000 to 180,000 between 2006 and 2012 in Cameroon with a total of 720,000 during the whole period. The change of the hosting institution in the case of REACH-PI prevented tracing the total investment during the period 2006–12, as from 2009 to 2012 the overall budget approximates US$ 640,000 non inclusive of the International Research Chair Initiative supporting the doctoral program in health policy and knowledge translation.

### Activities and outputs

Table 6 provides an account of the activities and outputs in terms of capacity building, knowledge management and linkage and exchange. The human capital for EIHSP was increased by more than thirty training workshops in Cameroon, Uganda and other countries (Kenya, Tanzania, Burkina Faso, Mali, Zambia, and Mozambique) to jointly build capacity for policymakers, researchers, civil society groups and media on EIHSP. Almost five hundred stakeholders were sensitized or trained by both KTPs including five Africans and four Canadians enrolled in the joint doctoral program in health policy and knowledge translation at Makerere University and McMaster University respectively.

**Table 6:** **KTP activities and outputs**

**Year**	**Functions**	**Activities and outputs**	
		**Cameroon**	**Uganda**
2006	Capacity building	EVIPNet workshop in Brazzaville. National EVIPNet Workshop. Application for a planning grant	Advocacy meetings with officials and global funders (IDRC, Swiss Tropical Institute, NOKC, AHPSR) to elicit support
	Knowledge management	Completion of the national health research system analysis	
	Linkage and exchange	Presentations with officials and funders to elicit support to the KTP	Presentations with officials to elicit support to the KTP
2007	Capacity building	Application to GHRI for a GHLA. Application to the EC-FP7 for the SURE project. SUPPORT workshop in Capetown	Application to the EC-FP7 for the SURE project
	Knowledge management	Stakeholder mapping and research mapping on nutrition and non communicable diseases	
	Linkage and exchange	Platforms bringing together actors in HIV/AIDS research, malaria research and Tuberculosis research, social sciences	
2008	Capacity building	Application to AHPSR for grant to support EIHP to transition towards a health SWAp. Executive Training for Research Application - EXTRA residency program. Addis workshop on writing policy briefs. Resource mobilization to establish an online repository for policy relevant health documents	Addis workshop on writing policy briefs
	Knowledge management	Preparation of the evidence brief on scaling up access to ACT	Preparation by REACH-PI EAC of two policy briefs on male circumcision and on scaling up access to ACT in Uganda and Tanzania
	Linkage and exchange	Policy dialogue on scaling up access to ACT	Policy dialogue on scaling up access to ACT
2009	Capacity building	Workshops for researchers on EIHP and evaluation of KTPs during the launch of SURE	Workshops for researchers on EIHP during the launch of SURE. The IDRC International Research Chair Initiative collaborative program for doctoral studies in health policy and knowledge translation is established between Makerere University and McMaster University
		Presentations with officials to elicit support to the KTP	
	Knowledge management	Establishment of a website providing access to online evidence resources. Preparation of two evidence briefs on strengthening community participation and community based health insurance	Preparation of the evidence brief on task shifting for maternal and child health
	Linkage and exchange	Priority exercise to identify priority topics. Policy dialogue on scaling up community based health insurance	Priority exercise to identify priority topics for evidence briefs
2010	Capacity building	Workshop for researchers to conduct Cochrane systematic reviews. Co-application for a DFID grant for the effective healthcare research with Stellenbosch University and South African Cochrane Centre	Launching of the collaborative program for doctoral studies in health policy and knowledge translation
	Knowledge management	Translation into French of abstracts of Cochrane systematic reviews. Preparation of three evidence briefs on reinforcing governance for health district development, reinforcing the health information systems for district servicing and scaling up malaria control interventions.	Preparation of an evidence brief on task shifting to optimize roles for mother and child health. Piloting of the rapid mechanism to respond to urgent needs of evidence of officials in the ministry of health
	Linkage and exchange	Presentations with officials in the ministry of health to elicit support. Two policy dialogues on governance for health district development and for scaling up malaria control interventions	Policy dialogue on task shifting to optimize the roles of healthcare providers for mother and child health. Rapid evidence syntheses to respond to health stakeholders’ urgent needs
2011	Capacity building	Workshops for policy makers, researchers, civil society representatives and media	Collaborative program for doctoral studies in health policy and knowledge translation
	Knowledge management	Preparation of evidence briefs on fixing the community health worker programme, retaining human resources for health in rural areas, scaling up enrolment in health insurance schemes	Rapid evidence syntheses to respond to health stakeholders’ urgent needs. Preparation of an evidence brief on skilled birth attendance
		Translation into French of abstracts of Cochrane systematic reviews. Preparation of bilingual evidence summaries	
	Linkage and exchange	Presentations with officials to elicit support to the KTP and researchers to engage into research synthesis. Policy dialogue to elicit the problem of human resources for health shortage in rural areas	Presentations with officials to elicit support to the KTP. Rapid evidence syntheses to respond to health stakeholders’ urgent needs
2012	Capacity building	Contribution to the development of the SURE Guides and Videos	Contribution to the development of the SURE Guides and Videos
	Knowledge management	Preparation of evidence briefs on increasing the coverage of antenatal care services, improving access to and quality of care in accident and emergency department in national and regional hospitals. Translation into French of abstracts of Cochrane systematic reviews. Preparation of bilingual evidence assessments. Rapid evidence syntheses to respond to health stakeholders’ urgent needs	Clearinghouse on health policy and systems research
			Rapid evidence syntheses to respond to health stakeholders’ urgent needs
			Preparation of a policy brief on palliative care
	Linkage and exchange	Presentations with officials from ministries of public health and social affairs and the University of Yaoundé 1 to elicit support Policy dialogues on retention of human resources for health in rural areas and improving coverage of antenatal care services and accident and emergency departments	Presentations with officials from the ministry of health to elicit support to the KTP
			Policy dialogues on skilled birth attendance
Total	Capacity building	Five successful grant applications. 16 capacity building workshops for policymakers, researchers and civil society groups and knowledge brokers in Cameroon, Burkina Faso, Kenya, Mali, Tanzania	Four successful grant applications. Capacity building workshops for policymakers, knowledge brokers and researchers in Uganda, Kenya, Tanzania, Burundi and Rwanda. Health policy PhD program. Contribution to SURE Guides and Videos
		Contribution to SURE Guides and Videos	
	Knowledge management	A functional website providing EIHP resources. 12 evidence briefs for policy. Six rapid evidence syntheses. SURE videos and guides available on-line.	A functional national clearinghouse on health policy and systems research
			Three evidence briefs for policy. 73 rapid evidence syntheses. SURE videos and guides available online
	Linkage and exchange	Seven evidence informed policy dialogues	Three evidence informed policy dialogues

Following the priority setting exercises, both KTPs have produced 15 evidence briefs for policy. Preparing evidence briefs was very labour intensive as few evidence briefs have required two full time equivalent knowledge brokers during one year. Between 2008 and 2012, EVIPNet Cameroon prepared 12 evidence briefs and REACH-PI Uganda prepared three evidence briefs. In line with the SURE grant plans, a mechanism to prepare rapid evidence syntheses in response to stakeholders’ urgent needs within days or weeks was piloted in Uganda starting 2010 and has required at least one full time equivalent knowledge broker. In 2012, the same mechanism was launched in Cameroon informed by the Uganda pilot. In total, six rapid evidence syntheses were prepared in Cameroon in 2012 versus 73 in 2010–2012 in Uganda. Under the effective health care research consortium collaboration with the SACC, EVIPNet Cameroon has prepared 12 bilingual summaries and translated into French 24 abstracts of Cochrane reviews in 2011–2012. Evidence products generally aligned with priorities to achieve health MDGs. EVIPNet Cameroon has maintained since 2009 a website providing access to evidence briefs and syntheses complementing the national electronic database of health documents housed by the division of health operations research. REACH-PI Uganda has established in 2012 a Uganda clearinghouse for health policy and systems research operating as a “one-stop shop” of health policy-relevant evidence. Informed by evidence gaps identified during the preparation of evidence briefs, both platforms applied for and received funds for building capacity to conduct policy relevant trials and Cochrane reviews in collaboration with the SACC. EVIPNet Cameroon has contributed to Cochrane Collaboration’s efforts to translate its products into French.

In terms of linkage and exchange, EVIPNet Cameroon organized seven policy dialogues informed by pre-circulated evidence briefs. The policy dialogues were jointly convened by the KTP secretariat and the Ministry of Health. The selection of participants was informed by the stakeholder analysis. Participants deliberated on scaling up access to artemisinin-based combination therapy (ACT), scaling up malaria control interventions, improving governance for health district development, retention of human resources for health in rural areas, scaling up community-based health insurance, improving antenatal care services coverage, improving access to and quality of care in the accident and emergency departments. REACH-PI Uganda organized three dialogues on scaling up access to ACT, task shifting for maternal and child health and, improving skilled birth attendance. EVIPNet Cameroon and REACH-PI Uganda played a central role organizing the first international forum on EIHP in LMICs (27–29 August 2012, Addis Ababa, Ethiopia) whose 121 participants were from 27 countries including 17 African countries. Participants were policymakers, international bureaucrats, knowledge brokers, researchers, civil society groups, and media.

### Stakeholders’ perspectives on the evidence briefs and policy dialogues

Table 7 summarizes the results of the survey of readers of ten evidence briefs. The respondents largely agreed that the briefs achieved their purpose of presenting the available research evidence on a high-priority policy issue in order to inform a policy dialogue where research evidence would be just one input to the discussion. The different design features of the brief were highly appreciated but respondents expressed lower satisfaction with the brief not concluding with any recommendations.

**Table 7:** **Summary of the evaluation of evidence briefs**

**Features of briefs produced**		**Cameroon (n = 99)**		**Uganda (n = 66)**	
		**Mean**	**SD**	**Mean**	**SD**
Overall assessment of satisfaction with the evidence briefs achieving its purpose		6.2	0.8	6.3	0.9
Design features of evidence briefs					
1.	Described the context for the issue being addressed	6.3	1.2	6.2	1.4
2.	Described different features of the problem, including (where possible) how it affects particular groups	6.1	1.2	6.0	1.4
3.	Described options for addressing the problem	6.0	1.1	5.8	1.4
4.	Described what is known, based on synthesized research evidence, about each of the options and where there are gaps in what is known	6.0	1.0	6.0	1.4
5.	Described key implementation considerations	6.1	1.1	6.0	1.3
6.	Employed systematic and transparent methods to identify, select, and assess synthesized research evidence	6.0	1.0	6.0	1.2
7.	Took quality considerations into account when discussing the research evidence	6.1	1.0	6.0	1.3
8.	Took local applicability considerations into account when discussing the research evidence	6.0	1.0	6.1	1.1
9.	Took equity considerations into account when discussing the research evidence	6.2	1.1	5.8	1.1
10.	Did not conclude with particular recommendations	5.4	1.3	5.4	1.9
11.	Employed a graded-entry format (e.g., a list of key messages and a full report)	6.4	1.0	6.2	1.2
12.	Included a reference list for those who wanted to read more about a particular systematic review or research study	6.4	1.0	6.3	1.7
13.	Was subjected to a review by at least one policymaker, at least one stakeholder, and at least one researcher (called a “merit” review process to distinguish it from “peer” review, which would typically only involve researchers in the review)	6.4	0.8	6.1	1.3

The ratings are on a Likert scale from 1 to 7 (least useful = 1 and most useful = 7) for question 1 to 13. The lowest rating (5.4) was for the briefs not concluding with particular recommendations. These are mean values for seven evidence briefs in Cameroon and three evidence briefs in Uganda.

Table 8 features the results of the survey of participants attending ten dialogues. All respondents felt the dialogues achieved their purpose of a full discussion of relevant considerations about a high-priority policy issue in order to inform action and the different features of how the dialogues were designed were considered very helpful including that the dialogue was informed by a pre-circulated evidence brief.

**Table 8:** **Summary of evaluation of deliberative dialogues**

**Features of dialogues convened by KTPs**		**Cameroon**		**Uganda**	
		**(n = 77; five dialogues)**		**(n = 69; three dialogues)**	
		**Mean**	**SD**	**Mean**	**SD**
Overall assessment		6.3	0.9	6.3	1.0
Design features commonly found in deliberative dialogues					
1.	Addressed a high-priority policy issue	6.6	0.9	6.4	1.2
2.	Provided an opportunity to discuss different features of the problem, including (where possible) how it affects particular groups	6.4	1.0	6.2	1.4
3.	Provided an opportunity to discuss options for addressing the problem	6.2	1.2	6.1	1.5
4.	Provided an opportunity to discuss key implementation considerations	6.2	0.9	6.1	1.3
5.	Provided an opportunity to discuss who might do what differently	6.4	0.9	5.7	1.3
6.	Was informed by a pre-circulated evidence brief	6.0	1.0	6.2	1.4
7.	Was informed by discussion about the full range of factors that can inform how to approach a problem, possible options for addressing it, and key implementation considerations	6.3	1.0	5.9	1.5
8.	Brought together many parties who could be involved in or affected by future decisions related to the issue	6.3	1.0	6.1	1.3
9.	Aimed for fair representation among policymakers, stakeholders, and researchers	6.3	0.8	6.2	1.2
10.	Engaged a facilitator to assist with deliberations	6.3	1.2	6.3	1.4
11.	Allowed for frank, off-the-record deliberations by following the Chatham House Rule	6.5	0.9	6.2	1.5
12.	Did not aim for consensus in the dialogue	6.3	1.1	6.2	1.3

The ratings were on Likert scales from 1 to 7 (least useful = 1 and most useful = 7) for question 1 to 12. The highest rating (6.6) was for the dialogue addressing a high-priority policy issue in Cameroon and the lowest rating (5.7) was for the dialogue providing an opportunity to discuss who might do what differently.

### Interpretive synthesis

Both initiatives are equipped with research units operating as national knowledge brokering institutions with regional influence. Within the two health systems, a network of local and global experts has created new spaces for inclusive evidence informed deliberations amongst policymakers, researchers and stakeholders on high-priority health policy topics related to MDGs. The interaction between the KTP secretariats and ministries of health and other stakeholders enabled the identification of priorities for evidence briefs as well as evidence gaps. Both initiatives have progressively expanded to cover the array of operations of a knowledge brokerage enterprise namely capacity building, knowledge management, and linkage and exchange. Applications to funders and advocacy meetings have enhanced their visibility and provided enabling resources towards institutionalization and sustainability.

The evidence briefs and rapid evidence syntheses prepared generally aligned with health policy and systems priorities to achieve the health MDGs. The technical and consensual natures of the topics addressed and the problem-driven approach have contributed to a high level of satisfaction amongst all categories of stakeholders. The mechanisms to address stakeholders’ urgent needs of evidence within days and weeks were well received. The briefs and syntheses have provided evidence-based problem frames, policy options and implementation strategies yielding potential changes in two of driving forces in policymaking namely interests and ideas.

This historical account illustrates how the convergence of local and global factors and agents has enabled the implementation of in-country efforts to support EIHSP related to health MDGs. It also illustrates how the differences in historical background, institutional anchorage, contexts and funding sources have led to differences in activities and outputs of these KTPs. The diversity of grant arrangements and the differences in institutional arrangements and planning cycles as well as the stability of health technocrats explain the differences in evidence outputs and the contrasted uptake of the rapid response mechanism. EVIPNet Cameroon was more prolific in preparing evidence briefs and organizing policy dialogues because of the closer ties with the ministry of health thus allowing working concurrently on several evidence briefs. REACH-PI Uganda prepared more rapid evidence syntheses and aligned the production of evidence briefs to the SURE project arrangements.

This historical account equally illustrates the unpredictability of the course of events during the initial decade of these initiatives conceived of as demonstration projects. Initial priority settings have been readjusted to align with changes in leadership within ministries of health and global funding opportunities (e.g. in Cameroon shifting from nutrition and chronic non communicable diseases to health district governance and health financing based on the grant from the AHPSR). Contributions from governments have remained in kind. In this documentary review, we failed to identify any empirical evidence on the influence or impact of the KTPs on the country climate for research use or on specific policy processes beyond the policy deliberations.

## Discussion

### Principal findings

The infrastructure, activities, outputs and outcomes of both initiatives encompass the full array of activities of knowledge brokerage enterprises and they have experimented at various levels the three key functions of such enterprise: capacity building, knowledge management and, linkage and exchange [8,28]. Indeed, both KTPs have trained almost 500 policymakers, researchers and stakeholders to facilitate researcher push and user pull [9]. More than 100 tailored evidence syntheses were produced, disseminated and made openly available online. Inclusive consultations were organized to identify high-priority policy issues related to health MDGs and structured stakeholder mappings laid the ground work to convene 10 deliberative dialogues informed by research evidence.

The historical account and the critical analysis of actors of these social experiments feature the influence of policy learning/diffusion in the establishment of policy networks and epistemic communities [31-33]. Leading researchers from northern universities linked with African researchers to create a new momentum for EIHSP, developing and testing new resources and tools to popularize knowledge translation activities across Anglophone and Francophone Africa. They exemplify the relevance of the recommendations formulated based on analysis of similar institutions in other settings [35].

### Strengths

This is the first historical account of what is constitutive of two KTPs housed in government institutions in sub Saharan Africa. It contributes empirical knowledge on the feasibility and practicality of enhancing the technical capacity of policymakers, researchers and stakeholders; preparing evidence briefs, syntheses and summaries; providing evidence related services; convening dialogues and creating space for evidence informed deliberations on high-priority health policy topics. This investigation complements the lessons learned from the Zambian Forum for Health Research (ZAMFOHR) case study [36], a national nongovernmental organization spearheading knowledge translation efforts in Zambia. It also enriches the recent gathering of lessons learned on KTPs by providing a longitudinal perspective on what constitutes a KTP and how it operates in two LMICs [18]. The study offers an insiders’ perspective as two authors have a deep knowledge and understanding of the context in Cameroon and Uganda and the authors have been involved with both KTPs from the inception phase through the current state of operations. This study also provides a grounded feedback to the chorus of voices calling for support to EIHSP and the recommended strategies for facilitating the uptake of research into policy in LMICs [37-42]. Finally, by providing an historical insight on institutional arrangements of KTPs, this study contributes empirical evidence to the call for new conceptual frameworks and methods to orient evaluation of efforts to support EIHSP in LMICs [20]. In that regard, the analytical framework used for this study lays the ground work for further political sciences informed perspectives on KTPs to comprehend their influence and impact.

### Limitations

This study presents three main limitations. First, the study is restricted to describing what is constitutive of the two KTPs since their inception; an assessment of their influence on specific policy processes and the climate for EIHP is still awaited to empirically inform the efforts to explicate KTPs using sound political sciences perspectives. Second relates to the nature of retrospective qualitative archival review, the exclusive use of official documents might have overlooked challenges experienced by the KTP implementers particularly the informal networks to navigate the health bureaucracies, to engage with officials and gain their support over time. The restriction to the KTPs’ archives might have prevented the capture of the external players’ views and perspectives and particularly the funding agencies. Last relates to the insiders’ narrative as recall bias and social desirability yield potential negative effects on neutrality and objectivity.

### Implications for local and global policymakers, stakeholders and researchers

This empirical documentation can inform the development of new initiatives with three implications: (i) those planning to establish initiative to support EIHSP in LMICs should carefully consider opportunities for national and international collaborations to mobilize political support from government officials and funding agencies; (ii) the critical role of participatory processes during priority setting exercises, stakeholders dialogue and needs assessment so as to secure commitment from both national policymakers and global players investing in health sector development; (iii) establishing an initiative to support EIHSP requires committed and skilled human resources to cope ably with intense and somehow stressful endeavour and to navigate the complex interfaces of knowledge to policy and action with a long term perspective.

This study equally provides a strong basis on which researchers can attune their efforts in developing and validating robust methods and tools to evaluate the effects and influence of KTPs [20].Indeed, the framework developed by Lavis and colleagues [9] to assess country efforts to link research to policy and used elsewhere [18] provide descriptive categories for efforts (e.g.; climate, research production, push efforts, facilitate user-pull, user-pull, exchange, and evaluation) engaged by a given country but doesn’t provide tools to assess the influence of such efforts. Further, the framework developed by Ward and colleagues [28] on what constitutes a knowledge brokering enterprise while accounting for the three main functions (e.g.; capacity building, knowledge management, and linkage and exchange) fails to account either for the effects and influence on drivers of policymaking (e.g.; institutions, interests and ideas) or the intersection with contextual factors during policymaking in environments permeate by cross jurisdictional learning. The need to have further reflection on the appropriate evaluative framework of KTPs remains valid [20].

The rising numbers of skilled individuals in EIHSP and the availability of contextualized evidence resources imply that national and global players investing in health sector development in Africa should create the enabling environment (e.g.; new rules and regulations, incentives) for and foster effective management and use of the human capital for policy analysis and research during health system planning and programming.

## Conclusion

This descriptive historical account of two KTPs housed in government institutions in Africa illustrates how the convergence of local and global factors and agents has enabled in-country efforts to support evidence-informed deliberations on high-priority health policy issues and lays the ground for further work to assess their influence on the climate for EIHSP and specific health policy processes.

## Abbreviations

EIHSP: Evidence informed health system policymaking
EVIPNet: Evidence informed policy network
KTP: Knowledge translation platform
LMIC: Low- and middle- income country
MDG: Millennium development goal
REACH-PI: Regional east african community health policy initiative
